# Perceptions of inclusion among students with and without self-reported learning disabilities in Saudi schools

**DOI:** 10.3389/fpsyg.2026.1820826

**Published:** 2026-08-07

**Authors:** Maha ALSulaiman, Umesh Sharma, Nourah Alshalhoub, Fiona May, Majed Alqahtani

**Affiliations:** 1 Department of Teaching and Learning, College of Education and Human Development, Princess Nourah Bint Abdulrahman University, Riyadh, Saudi Arabia; 2 Vice Provost for People and Culture, University of Nottingham Ningbo China, Ningbo, China; 3 Monash University, Melbourne, VIC, Australia; 4 Ministry of Education, Saudi Arabia Ministry of Education, Riyadh, Saudi Arabia

**Keywords:** inclusion climate, inclusive education, Saudi Arabia, specific learning disabilities, student perceptions

## Abstract

**Introduction:**

This study examined perceptions of inclusive education among Saudi students with and without self-reported indicators of Specific Learning Disabilities (SLD), addressing limited evidence from Saudi schools.

**Methods:**

A correlational design was used with 694 Saudi students in Grades 4–9. Participants completed Arabic-translated and culturally adapted measures of inclusion climate and self-reported SLD indicators. Descriptive statistics, Cronbach's alpha, confirmatory factor analyses (CFA), Pearson correlations, and independent-samples *t*-tests were conducted.

**Results:**

Higher self-reported SLD indicators were significantly associated with lower perceived inclusion. Female students and those accessing resource rooms reported higher SLD indicators, whereas perceived inclusion did not differ by gender or resource room access. Elementary students reported higher perceived inclusion than middle school students.

**Discussion:**

The findings highlight developmental differences in inclusion experiences and underscore the importance of fostering belonging in inclusive schools. They also provide practical implications for strengthening inclusive practices and promoting equitable participation in mainstream education.

## Highlights

- This study examined perceptions of the inclusive education climate among students with and without self-reported indicators of Specific Learning Disabilities (SLD) in Saudi Arabian schools.
- Students who reported greater indicators of SLD also reported feeling less included in their school environment.
- Female students and those accessing resource room support provided higher levels of self-reported indicators of SLD, while elementary students reported higher inclusion than middle school students.
- Findings underscore the need for inclusive policies and practices that promote belonging, participation, and equitable support for all learners.

## Introduction

Despite growing global interest in inclusive education, little is known about how Saudi Arabian students with and without self-reported Specific Learning Disabilities (SLD) perceive the inclusion climate of their schools. Moreover, although international studies highlight the importance of students' lived experiences of inclusion (e.g., classroom belonging, peer support, and perceived fairness), few studies have examined these perceptions in Middle Eastern contexts. The current study aims to address this gap.

The global importance of inclusive education is highlighted by the signing of the Salamanca Statement and Framework for Action on Special Needs Education (UNESCO, 1994) and the widespread ratification of the United Nations (2007). Although early efforts focused primarily on students with disabilities, contemporary interpretations of inclusive education now extend to students from diverse economic, linguistic, social, and cultural backgrounds. Internationally, the presence of diverse learners, including those with disabilities, has increased significantly in mainstream schools (Forlin et al., 2013). Despite notable advances in policy frameworks and legislative commitments aligned with the United Nations Sustainable Development Goals (SDGs), particularly Sustainable Development Goal 4 (Quality Education), which aims to ensure inclusive and equitable quality education and promote lifelong learning opportunities for all, many education systems continue to face challenges in translating inclusive policies into effective school practices (United Nations, 2016, 2023), many education systems continue to face challenges in translating inclusive policies into effective school practices. These challenges have been documented across a range of countries, including Australia, the United States, and various European contexts (e.g., Florian and Black-Hawkins, 2011; Messiou, 2019).

The Saudi Arabian Ministry of Education (MOE) took its first major step toward inclusion by introducing inclusive practices in 1994, and since then, meaningful expansions have been made in both the availability and quality of support for students with disabilities. The Kingdom's commitment is reflected in its allocation of 19% of the national budget to education in 2020, surpassing the OECD average of 11% (OECD, 2020). In recent years, enhancements to teacher training in both special and inclusive education, alongside ongoing curriculum modernization and the planned integration of professional teaching standards and licensing, demonstrate alignment with the United Nation's Sustainable Development Goal 4: quality Education—particularly its call to “ensure inclusive and equitable quality education and promote lifelong learning opportunities for all.” Cultural attitudes have also begun to shift, with evidence of increasing public awareness and acceptance of individuals with disabilities (Aldabas, 2015). This movement is reinforced through Vision 2030, which emphasizes that people with disabilities should “receive the education and job opportunities that will ensure their independence and integration as effective members of society” (OECD, 2020; Saudi Vision 2030, 2016; United Nations, 2023). Comparable large-scale reform movements aimed at strengthening inclusive systems have also been reported in countries such as Qatar, the United Arab Emirates, and Singapore (e.g., Gaad, 2010; Forlin, 2008).

A significant milestone was reached in August 2023 when the Council of Ministers approved the Rights of Persons with Disabilities Act, replacing the prior Disability Code. The Act emphasizes independence, inclusion, and empowerment and is closely aligned with the United Nations Convention on the Rights of Persons with Disabilities, ratified by Saudi Arabia in 2008 (Authority of People with Disability, 2023).

Against this backdrop, the present study examines students' perceptions of inclusion in Saudi schools, focusing on differences between students with and without SLD to generate evidence that informs both research and practice in inclusive education.

### Inclusion climate

Given the considerable variability in how learning disabilities manifest across individuals, effective support requires flexible, individualized, and context-responsive approaches that address students' unique educational needs (Sainz et al., 2025). In addition, many students with learning disabilities report challenges in developing and maintaining positive peer relationships and may experience marginalization or social exclusion, which can negatively affect their sense of belonging within the school community (Gal and Ryder, 2025). Academic disabilities are often accompanied by social and emotional challenges, including low self-esteem, anxiety, academic stress, and symptoms of depression (Bergey et al., 2018; Vuori et al., 2008).

Taken together, these interconnected academic, social, and emotional challenges underscore the importance of inclusive educational practices that foster meaningful participation, engagement, and a sense of belonging for all learners within the school environment (Woodcock et al., 2022).

Central to the operationalization of these inclusive practices is the concept of inclusion climate, which has received increasing attention in the literature. Mitchell et al. (2010) defined school climate as “the shared beliefs, values, and attitudes that shape interactions between students, teachers, and administrators” (p. 272). Drawing on this foundational definition, researchers have investigated the characteristics that cultivate a positive inclusion climate (Schwab et al., 2018). Factors most frequently identified include teacher and school leader attitudes toward inclusion, the influence of school culture and policies, peer relationships and support, and the use of instructional approaches such as differentiation and personalization. Such elements contribute to environments in which students feel connected, valued, and supported (Bossaert et al., 2013; De Vroey et al., 2016).

A growing body of research indicates that inclusive climates shape peer attitudes toward students with disabilities. When students without disabilities experience empathy, fairness, and support in their classrooms, they are more likely to hold positive attitudes toward peers with disabilities, often mediated by classroom climate (Zurbriggen et al., 2021). Similarly, Fu et al. (2022) found that students generally demonstrated neutral attitudes toward peers with disabilities, with girls reporting more positive attitudes than boys. Inclusive climate, empathy, and attitudes toward classmates with disabilities were positively associated. Consistent with these findings, Mirete et al. (2020) identified empathy as a significant predictor of positive attitudes toward individuals with intellectual disabilities. Students with higher levels of empathy were more likely to demonstrate accepting and inclusive attitudes, whereas contact or previous experience alone was not always sufficient to produce positive outcomes. Furthermore, Rademaker et al. (2020) suggested that the effectiveness of contact-based strategies depends on the nature of the intervention, with integrated activities and playgroups producing the most favorable outcomes. In contrast, cooperative learning and peer assistance did not consistently result in more positive attitudes toward students with disabilities (Poikola et al., 2024).

Additionally, researchers emphasize the importance of student-centered perceptions of climate, as students' perceptions are consistently associated with social and academic outcomes and reflect everyday relational experiences that may not be evident in teacher or observer reports (Mitchell et al., 2010; Schwab et al., 2018). Similar findings have been reported across European, Asian, and North American contexts, highlighting the cross-cultural relevance of inclusion climate and students' perceptions of inclusive educational experiences (Bear et al., 2018; Schwab et al., 2018).

### Including student voices to assess inclusion climate

Student voice has become a substantial focus in educational research, grounded in a commitment to recognize students, particularly those from marginalized groups, as active contributors to decisions affecting their learning (Cook-Sather, 2006; Fielding, 2004). The premise is that students, as primary recipients of educational services, can provide essential insights into teaching practices, school environments, and inclusive approaches (Nelson and Christensen, 2009; Schwab et al., 2018). Connor and Cavendish (2020) highlighted “the power of student voice” (p. 288), emphasizing that involving students in discussions about their education can foster agency, empowerment, and improved academic outcomes (Barile et al., 2012; Lundy, 2007).

Recent evidence further reinforces the importance of understanding inclusion from students' perspectives. Tsibidaki (2026) found that students with learning disabilities viewed participation and belonging as closely linked to academic accessibility, individualized support, and positive relationships with teachers and peers. Likewise, Poikola et al. (2025) reported that students perceived inclusive learning environments as most effective when characterized by peer support, positive group dynamics, and supportive teacher practices, whereas bullying, noise, and insufficient support undermined participation. Together, these studies indicate that meaningful inclusion extends beyond physical placement in mainstream classrooms and is strongly influenced by the social, relational, and instructional features of the learning environment. They also highlight the value of student voice in informing inclusive policy and practice.

In the broader scholarly literature, student voice is conceptualized as a continuum from consultation, to participation, to genuine partnership with adults (e.g., Lundy, 2007; Mitra, 2004). Importantly, student voice can be captured through multiple methodological approaches, including large-scale self-report surveys that systematically document students' perceptions of belonging, participation, and support within their learning environments (Schwab et al., 2018; Bear et al., 2018). In the present study, student voice is operationalized at a consultative level through students' self-reported perceptions of inclusion climate, rather than through in-depth qualitative or participatory methods. This approach prioritizes breadth and comparability across a large sample with the intent of providing valuable evidence from students with and without self-reported indicators of SLD whose perspectives are seldom documented in the region, while acknowledging that it does not provide the same depth and nuance as qualitative designs.

The value of student voice in inclusive education is recognized globally, though context-specific approaches are essential. In contexts where inclusive education is still emerging, student perspectives are critically important for informing effective policy and practice (Alnahdi, 2020; Alquraini, 2011). Vaughn and Klingner (1998) note that despite ongoing debates concerning the inclusion of students with learning disabilities, the voices of these students remain largely absent from research and decision-making.

### Saudi Arabian context

The percentage of people with disabilities in Saudi Arabia is 5.9% (General Authority for Statistics, 2022). Motor disabilities are most prevalent (1.0%), followed by visual and communication disabilities, each affecting 0.6% of the total population (General Authority for Statistics, 2023). These national estimates may under-represent less visible or variably identified disabilities, including learning disabilities, due to differences in diagnostic practices, access to assessment services, and reporting systems.

Recent national strategies and policies, including the National Strategy for the Development of General Education and Vision 2030, have emphasized equitable learning opportunities for all students (Alsolami, 2024). Public schools serve over 80% of students across the Kingdom, and the proportion of students in special education has remained steady since 2014 (Almalky and Alwahbi, 2023). However, there is evidence of increasing numbers of students with disabilities, particularly those with learning and physical disabilities, entering general education settings (Almalky and Alwahbi, 2023; Gibbs and Bozaid, 2022). According to MOE statistics (Ministry of Education, 2024), students with SLD represent approximately one-third (32.6%) of all students with special needs. Comparable prevalence patterns are documented internationally (National Center for Education Statistics, 2024).

SLD are neurodevelopmental disorders characterized by significant, measurable difficulties in reading, writing, or mathematics (American Psychiatric Association, 2013). These difficulties are not attributable to intellectual disability, neurological disorders, or broader developmental delays (Bonifacci et al., 2016). Without adequate interventions and accommodations, SLD can impede academic achievement and daily functioning. Because formal diagnostic verification is not always feasible in large school-based studies, self-report screening tools are sometimes used to capture students' perceived learning disabilities and related indicators, while recognizing these tools do not confirm diagnosis (Smythe and Everatt, 2001).

Despite progress, inclusion in Saudi Arabia remains shaped largely by a medical model of disability, raising questions about the extent to which students experience meaningful inclusion (Aldabas, 2015; Al-Mousa, 2010; Madhesh, 2023). Indeed, research indicates that physical placement in mainstream classrooms does not automatically guarantee social inclusion. Students with special educational needs (SEN) often encounter challenges related to peer acceptance and social participation, which can contribute to experiences of loneliness and exclusion (Avramidis and Aroni, 2020; Toulia et al., 2022). Consequently, inclusion should be conceptualized not merely as a matter of spatial placement, but as a multidimensional process shaped by everyday interactions and the broader school culture.

This issue is particularly relevant for students with specific learning disabilities (SLD), most of whom attend mainstream classrooms while receiving additional support in resource rooms. In this model, qualified special education teachers provide targeted interventions during part of the school day. Although this approach resembles pull-out support systems commonly used internationally, its effectiveness depends on several factors, including the quality and intensity of instruction, alignment between resource-room and classroom curricula, collaboration between general and special educators, and the extent to which withdrawal from the general classroom influences students' participation, peer relationships, and sense of belonging (McLeskey et al., 2014).

### Current study

This study aimed to explore the experiences of Saudi Arabian students with and without self-reported indicators of SLD regarding their school inclusion climate. Specifically, we addressed the following research questions:

1. What are the psychometric properties of the Inclusion Climate Scale and the Self-Reporting Indicators of a Specific Learning Disability Scale for Saudi Arabian students?
2. Is there a relationship between self-reported indicators of SLD and student perceptions of inclusion?
3. Are there differences in self-reported indicators of SLD according to demographic characteristics, including gender, school level, and whether a student is receiving resource room support?
4. Are there differences in student perceptions of inclusion according to demographic characteristics, including gender, school level, and whether a student is receiving resource room support?

## Methods

### Participants

Schools were selected using a convenience sampling approach based on accessibility and willingness to participate. No targeted oversampling of students receiving resource room support was undertaken; the proportion of students reporting such support reflects the composition of participating schools. The sample included both public and private schools across multiple areas of Riyadh, capturing variation in school contexts; however, in the Saudi context, support services and educational practices for students with learning disabilities are broadly comparable across the two sectors, although class sizes may vary. The relatively large sample size and inclusion of schools from different areas within Riyadh enhance the potential generalizability of the findings, although caution is warranted when extending the results beyond the immediate study context.

Participants included 694 students recruited from 24 public and private schools in Riyadh District of Saudi Arabia. Approximately two-thirds of the sample were female (*n* = 457; 65.85%), and 237 (34.15%) were male. Students were drawn from 6 year levels, ranging from Grade 4 (*n* = 109; 15.79%) to Grade 9 (described in Saudi Arabia as Third Intermediate Level; *n* = 145; 21.01%). In the Saudi school system, elementary school comprises Grades 1–6, while intermediate school comprises Grades 7–9; these levels represent transitions from foundational to more subject-specialized learning. Students from Grades 4–9 were included because this age range represented students considered developmentally able to provide meaningful self-reported information about their learning experiences and perceptions of inclusion. Although students across these grades differ in academic demands and educational experiences, the constructs examined in the present study (perceived inclusion climate and self-reported indicators of learning disabilities) are relevant across both elementary and intermediate school contexts. Potential developmental differences were therefore considered by examining school level as a demographic variable in the analyses.

The average age of participants was 12.14 years (SD = 1.81). Of the total sample, 258 students (39.15%) reported receiving additional learning support through their school's resource room, which are specialized environments staffed by teachers who provide targeted instruction for students with learning disabilities. The demographic characteristics of participants are presented in Table 1.

**Table 1:** Demographic characteristics of participants.

Characteristic	*n*	%	N^*^
Student gender			694
Male	237	34.15%	
Female	457	65.85%	
Student grade			690
4	109	15.79%	
5	117	16.95%	
6	137	19.85%	
First intermediate	121	17.53%	
Second intermediate	61	8.84%	
Third intermediate	145	21.01%	
Resource room			659
Yes	258	39.15%	
No	401	60.85%	
Student age	*M* = 12.14 years; *SD* = 1.81	678	

^*^The Ns reported in this table differ slightly across categories due to non-response from some students on individual items.

### Measures

Data were collected using a three-part survey including (1) demographic information, (2) the Self-Reporting Indicators of a Specific Learning Disability Scale (Smythe and Everatt, 2001), and (3) the Inclusion Climate Scale (ICS; Schwab et al., 2018).

#### Demographic information

Demographic data included gender, age, grade level, school type, geographical district, and whether the student received additional support via the school's resource room. Where applicable, students were also asked whether they had ever been formally identified by a specialist or teacher as having learning difficulties. While the survey did not collect formal diagnostic reports, students who reported receiving resource room support would typically have been identified by the school as having learning disabilities, including Specific Learning Disabilities (SLD). In Saudi Arabia, resource room eligibility commonly reflects teacher or specialist identified concerns in literacy, numeracy, organization or attention. However, SLD status was not verified through school records or formal diagnostic documentation for the purposes of this study. Therefore, resource room access is treated as an indicator of receiving additional learning support rather than confirmation of a clinically diagnosed SLD.

#### Part One: Inclusion Climate Scale (ICS)

Part One included the 28-item Inclusion Climate Scale (Schwab et al., 2018), which measures students' perceptions of inclusion across dimensions such as presence, participation, acceptance, achievement, belonging, and satisfaction with schooling. The ICS has demonstrated good internal consistency (α = 0.89; Schwab et al., 2018). In the present study, the scale was translated and culturally adapted for use in the Saudi context. Internal consistency was examined to assess reliability and confirmatory factor analyses were conducted to examine the extent to which the structure of the translated scale aligned with the original instrument.

Items were translated into Arabic and then independently back-translated into English to ensure semantic equivalence. To strengthen cultural and linguistic validity, the translated version was reviewed by three specialists in inclusive education. To support comprehension for students with potential reading or language-processing disabilities, items were reviewed for age-appropriate wording during expert review, and administration procedures permitted items to be read aloud verbatim when requested. This process aimed to reduce construct-irrelevant variance due to decoding demands while maintaining consistency in item wording. A three-point Likert scale was used (1 = No, 2 = Sometimes, and 3 = Yes), with higher scores indicating stronger perceptions of inclusion.

Although Schwab et al. (2018) reported confirmatory factor analysis (CFA) results in their original validation, the present study focused on the translation and cultural adaptation of the scale for use in the Saudi context. Consistent with recommendations for cross-cultural adaptation, translation, expert review, and internal consistency were prioritized as initial steps in establishing measurement suitability (Hambleton et al., 2005). In addition, confirmatory factor analyses were conducted to examine the extent to which the structure of the translated scale aligned with the original instrument. These analyses provide initial evidence of construct validity in this context, however, further research is needed to examine measurement invariance and the stability of the factor structure across diverse samples. The primary aim of the current study was not to re-validate the instrument in full, but to examine its utility for capturing student perceptions of inclusion and self-reported learning difficulties in a previously under-researched context.

#### Part Two: Self-Reporting Indicators of a Specific Learning Disability Scale

Part Two included the 15-item Self-Reporting Indicators of a Specific Learning Disability Scale (Smythe and Everatt, 2001). Items assess literacy skills, word-finding, and organizational difficulties associated with SLD. Responses are rated on a four-point Likert scale (1 = Rarely, 2 = Occasionally, 3 = Often, and 4 = Most of the time), with total scores computed by summing all items. It is important to note that this instrument captures self-reported indicators associated with learning disabilities and does not constitute a diagnostic assessment of Specific Learning Disabilities.

As with the ICS, the scale was translated into Arabic and back-translated into English to ensure meaning was preserved. The scale is widely used internationally as a screening instrument and demonstrates strong reliability. Internal consistency (Cronbach's alpha) and CFA were conducted to examine the reliability and underlying structure of the translated scale. During administration, educators' assistants were available to support students, particularly those with reading disabilities, by reading items aloud or clarifying wording when required. Assistants were trained to read items aloud verbatim, repeat items upon request, and clarify individual words using neutral definitions without providing examples, interpretations, or suggestions that could cue particular responses. They were instructed not to paraphrase items, comment on responses, or indicate preferred answers. Support was limited strictly to facilitating access and comprehension. This ensured equitable access and mitigated the possibility that lower scores may have reflected difficulties decoding the survey language.

### Procedure

Ethical approval was obtained from the Institutional Review Board (IRB) at the university and the Saudi Ministry of Education. Prior to data collection, all survey tools were translated and back-translated, then reviewed by experts in inclusive education and special education to confirm linguistic accuracy and cultural suitability.

Twenty-four schools were invited to participate, representing a mix of public and private institutions and including equivalent numbers of boys' and girls' schools, in line with Saudi Arabia's gender-segregated educational structure. Schools were selected to ensure diversity in geographical location and grade levels served. Schools educating only early primary students were excluded because some questionnaire items were considered too cognitively demanding for very young learners.

Written informed consent was obtained from students and their guardians. Surveys were administered during school hours, with trained educators' assistants supporting completion where necessary. For students with self-reported SLD, assistants ensured comprehension without influencing responses, consistent with recommended ethical procedures for inclusive research involving children with learning difficulties. To minimize inadvertent influence, assistants followed a brief standardized support protocol and were instructed to provide the same type of assistance across all students requiring help (e.g., verbatim reading and neutral clarification only).

### Data analysis

Descriptive statistics were calculated for all demographic variables. Preliminary tests confirmed that assumptions of normality, linearity, and homoscedasticity were not violated. Scale means, standard deviations, and internal consistency (Cronbach's alpha) were calculated for the ICS and the Self-Reporting Indicators of a Specific Learning Disability. Confirmatory factor analyses (CFA) were conducted using maximum likelihood estimation to examine the factor structure of the translated instruments. Model fit was evaluated using multiple indices, including the Comparative Fit Index (CFI), Tucker–Lewis Index (TLI), Root Mean Square Error of Approximation (RMSEA), and Standardized Root Mean Square Residual (SRMR), in line with established recommendations.

Pearson product-moment correlations were used to examine associations between inclusion climate scores and self-reported SLD. Finally, a series of independent samples *t*-tests were conducted to compare differences in ratings of inclusion climate and self-reported specific learning disability for males as compared to females; for students in elementary school as compared to middle school; and for students receiving support in the school's resource room compared to students not receiving resource room support. This analytic approach aligned with the study's exploratory aims and provides a clear framework for examining group differences relevant to inclusive education research. Sample sizes varied slightly across analyses due to item-level non-response. Cases with missing data on variables relevant to a given analysis were excluded using listwise deletion. The reduction in sample size for the correlation analysis reflects missing responses on one or both of the key variables. No imputation procedures were applied, and the analyses are therefore based on available data for each comparison. Given the low proportion of item-level non-response, missing data were not expected to have materially influenced the study findings.

## Results

The purpose of this study was to examine the extent to which students with and without self-reported indicators of Specific Learning Disabilities (SLD) experience an inclusive school environment, and to explore whether perceptions of inclusion differ by gender, school level, or access to additional learning support.

### Psychometric properties of the Inclusion Climate Scale and the Self-Reporting Indicators of a Specific Learning Disability Scale

Scores on the Arabic-translated Inclusion Climate Scale (ICS) ranged from 28 to 84, with higher scores indicating more positive perceptions of inclusion. Internal consistency for the ICS was strong, with a Cronbach's alpha of 0.86.

Scores on the Arabic-translated Self-Reporting Indicators of a Specific Learning Disability Scale ranged from 15 to 60, with higher scores reflecting stronger self-reported indicators of SLD. Internal consistency for this scale was adequate (α = 0.73). These results indicate that both translated instruments demonstrated acceptable reliability for use in this context.

Confirmatory factor analyses were conducted to examine the factor structure of the translated instruments. For the 28-item Inclusion Climate Scale, both a one-factor model and a theoretically informed two-factor model were tested. The two-factor model, reflecting Teacher Support and Care and Emotional Experience, demonstrated improved fit relative to the one-factor model, χ^2^_(349)_ = 936.68, *p* < 0.001, CFI = 0.814, TLI = 0.798, RMSEA = 0.056, and SRMR = 0.056. The one-factor model showed weaker fit, χ^2^_(350)_ = 1,031.73, *p* < 0.001, CFI = 0.784, TLI = 0.767, RMSEA = 0.061, and SRMR = 0.058. Overall, these findings provide support for the two-factor structure of the Inclusion Climate Scale, consistent with the original conceptualization of the instrument (Schwab et al., 2018), although incremental fit indices were below conventional thresholds.

For the Self-Reporting Indicators of a Specific Learning Disability Scale, a one-factor model was tested. Model fit was mixed, χ^2^_(90)_ = 482.07, *p* < 0.001, CFI = 0.802, TLI = 0.769, RMSEA = 0.088, and SRMR = 0.084, suggesting partial support for a unidimensional structure in the present sample.

Although both scales demonstrated acceptable internal consistency, findings should be interpreted alongside the CFA results, which provide initial support for the construct validity of the translated instruments. Further validation research across different populations and educational contexts is warranted to confirm the stability of the factor structures.

### Relationship between self-reported indicators of SLD and student perceptions of inclusion

A Pearson product-moment correlation examined the association between students' self-reported indicators of SLD and their perceptions of inclusion. Results indicated a small but statistically significant negative correlation, *r* = −0.19, *n* = 533, and *p* < 0.001, meaning that higher levels of self-reported indicators of SLD were associated with lower ratings of inclusion (see Table 2). The relationship between self-reported indicators of SLD and inclusion climate scores shows a slight downward trend, indicating that students who reported greater learning difficulties tended to report lower levels of perceived inclusion. While the association is modest in magnitude, the pattern is consistent across the distribution of scores.

**Table 2:** Summary of group differences in self-reported indicators of SLD and inclusion climate.

Outcome variable	Group comparison	Significant difference?	Direction of difference	Effect size (η^2^)
Self-reported specific learning disability	Female vs. male	Yes	Females > males	0.02 (small)
	Elementary vs. Middle	No	–	–
	Resource room vs. no resource room	Yes	Resource room > no resource room	0.02 (small)
Inclusion climate (ICS)	Female vs. male	No	–	–
	Resource room vs. no resource room	No	–	–
	Elementary vs. middle	Yes	Elementary > middle	0.07 (moderate)

Significance was determined using independent samples t-tests with a Bonferroni-adjusted α = 0.01 to control for Type I error across multiple comparisons. Effect sizes are interpreted using Cohen's (1988) conventions.

### Differences in self-reported indicators of SLD according to demographic characteristics

Overall, self-reported indicators of SLD fell in the mid-range (*M* = 29.84, *SD* = 7.46) on a scale with possible scores from 15 to 60. Independent samples *t*-tests were conducted to examine differences across gender, school level, and resource room support. A Bonferroni adjustment yielded a revised alpha level of 0.01 to control for Type I error. A summary of these findings and group comparisons is presented in Table 2.

#### Gender

There was a statistically significant difference in self-reported indicators of SLD between male and female students, *t*_(565)_ = −3.45, *p* < 0.001, with females reporting higher levels (*M* = 30.62, *SD* = 7.35) than males (*M* = 28.37, *SD* = 7.46; see Table 2).The effect size (η^2^ =0.02) indicated a small effect (Cohen, 1988).

#### School level

There was no significant difference between elementary school students (*M* = 30.37, *SD* = 7.69) and middle school students (*M* = 29.31, *SD* = 7.20) on self-reported SLD.

#### Resource room access

A statistically significant difference emerged for resource room use, *t*_(538)_ = 3.55, *p* < 0.001, with students who received resource room support providing higher self-reported SLD indicators (*M* = 31.21, *SD* = 7.88) compared with those who did not (*M* = 28.90, *SD* = 7.02; see Table 2). This effect size (η^2^ =0.02) also represented a small effect.

### Differences in student perceptions of inclusion according to demographic characteristics

Students' perceptions of inclusion were generally positive (*M* = 72.75, *SD* = 8.15), on a scale ranging from 28 to 84 (see Table 2).

#### Gender

No significant differences were found between males (*M* = 72.39, *SD* = 9.01) and females (*M* = 72.93, *SD* = 7.69) in their perceptions of inclusion in this sample, which may reflect limitations in measurement sensitivity or sample characteristics rather than the absence of meaningful differences.

#### Resource room access

There were no significant differences in inclusion ratings between students receiving support (*M* = 72.68, *SD* = 8.12) and those not receiving support (*M* = 72.58, *SD* = 8.19), which again may be an artifact of measurement sensitivity or sample characteristics.

#### School level

A significant difference was observed according to school level, *t*_(436)_ = 6.16, *p* < 0.001, with elementary school students reporting higher perceptions of inclusion (*M* = 74.73, *SD* = 6.78) than middle school students (*M* = 70.40, *SD* = 8.99; see Table 2). The effect size (η^2^ =0.07) suggested a moderate effect (Cohen, 1988).

## Discussion

The findings from this study provide important insights into how students with and without self-reported indicators of Specific Learning Disabilities (SLD) perceive the inclusion climate within Saudi schools. The Arabic-translated Inclusion Climate Scale and the Self-Reporting Indicators of a Specific Learning Disability Scale demonstrated satisfactory internal consistency, in line with recommendations highlighting the importance of reliable and culturally responsive measurement tools in educational research (Taber, 2018; Tavakol and Dennick, 2011). Confirmatory factor analyses provided initial evidence of construct validity, with support for the two-factor structure of the Inclusion Climate Scale and partial support for a unidimensional structure of the SLD indicators scale. These findings reflect broader calls for valid cross-cultural measurement in inclusive education research (Artiles and Kozleski, 2016).

While several statistically significant findings emerged, some associations and group differences were characterized by small effect sizes. Accordingly, these findings should be interpreted as indicating meaningful patterns that warrant further investigation rather than as evidence of substantial differences between student groups.

A key finding was the significant negative correlation between self-reported indicators of SLD and perceptions of inclusion. Rather than merely indicating a statistical relationship, this finding suggests that students who experience self-reported learning disabilities may encounter barriers not only to academic access but also to social participation and a sense of belonging. This interpretation aligns with international research demonstrating that students with disabilities often experience barriers to full participation and belonging (Murray and Greenberg, 2006). From a theoretical perspective, this supports inclusive education frameworks that conceptualize inclusion as both a structural and relational process. Although the effect size was small, its consistency with global trends suggests a modest association that may have practical relevance, although the small effect size indicates that this relationship should be interpreted with caution. As (Florian and Black-Hawkins 2011) argue, inclusive education must extend beyond access to mainstream classrooms and intentionally foster meaningful participation and social acceptance. Importantly, the present findings reinforce the need for schools to move beyond placement-based definitions of inclusion and prioritize meaningful participation and student voice.

The finding is also consistent with concerns raised about the limitations of pull-out support models. Vaughn and Klingner (1998) highlighted that while resource rooms may offer targeted academic instruction, they can inadvertently reduce students' sense of belonging and increase feelings of separation from peers. Research also demonstrates that physical presence alone does not ensure social acceptance or emotional inclusion (Prince and Hadwin, 2013).

Taken together, these findings suggest that inclusive practice should increasingly emphasize in-class support models and collaborative teaching approaches that minimize social separation, while also underscoring the importance of examining inclusion through the lens of student voice. As Baroutsis et al. (2016) noted, failing to incorporate students' perspectives can perpetuate patterns of alienation and marginalization, particularly for students with disabilities. Consistent with this, Connor and Cavendish (2020) emphasized the need to create classroom environments that are intentionally designed to support student engagement, agency, and positive peer relationships.

### Gender, resource room use, and school level differences

Gender differences emerged in self-reported SLD, with female students reporting higher levels of difficulty than males. Although the effect size was small, this pattern mirrors research suggesting that gender influences academic self-perception, help-seeking behaviors, and self-reporting of learning challenges (Duckworth and Seligman, 2006; Voyer and Voyer, 2014). This pattern may reflect differences in self-awareness, reporting tendencies, or social expectations rather than actual differences in ability. From a practical standpoint, these findings highlight the importance of developing gender-sensitive identification and support practices within schools, as well as the need for further investigation into gendered dynamics in the identification of learning needs and provision of support.

Students who accessed the resource room reported higher levels of self-reported indicators of SLD, as expected given the purpose of these programs. However, it remains unclear whether these perceptions reflect pre-existing learning difficulties or are influenced by the receipt of support, raising important questions about potential labeling effects and their impact on students' self-perceptions. In Saudi Arabia, resource rooms constitute a central component of support for students with SLD; however, research across multiple contexts suggests that more integrated, classroom-based models may reduce stigma and enhance inclusion (Hehir, 2002). Consistent with international trends, there is a growing shift toward flexible support structures that minimize reliance on withdrawal approaches (e.g., McLeskey et al., 2014), indicating that schools may benefit from adopting more inclusive, in-class support practices. In addition, gender differences emerged, with female students reporting higher levels of difficulty. This pattern may reflect differences in self-awareness, reporting tendencies, or social expectations rather than actual differences in ability, highlighting the need for gender-sensitive identification and support practices within schools.

### Differences in perceptions of inclusion across school levels

A significant difference was observed in perceptions of inclusion between elementary and middle school students, with younger students reporting higher levels of inclusion. A significant difference was observed in perceptions of inclusion between elementary and middle school students, with younger students reporting higher levels of inclusion. This pattern may reflect developmental changes in peer dynamics, academic pressures, and social experiences during early adolescence, and aligns with research documenting declines in school connectedness, perceived social support, and engagement at this stage (Eccles et al., 1993; Wigfield and Eccles, 2000). The moderate effect size highlights middle school as a critical period for targeted inclusive interventions. Consistent with this, Vaughn and Klingner (1998) found that while younger students with disabilities may prefer in-class support, older students often view resource rooms more favorably, yet, simultaneously experience social stigma associated with their use. These developmental differences underscore the importance of tailoring inclusive practices across school phases.

### Policy implications

The negative association between self-reported SLD and perceptions of inclusion underscores the need for schools to adopt practices that promote both academic and social inclusion. Drawing from (Florian and Black-Hawkins 2011), inclusive education should prioritize belonging, participation, and recognition of student diversity, not solely academic accommodations.

Gender differences in self-reported SLD highlight the importance of carefully monitoring referral practices, self-perception patterns, and how students access support. Educators should be attentive to potential gender biases in identification and ensure equitable access to learning supports.

Consistent with Al-Muntashari et al. (2024), fostering collaboration between teachers, strengthening communication with parents, and developing individualized support strategies, such as personalized education plans, will be critical for enhancing inclusion consistent with recent evidence highlighting the importance of coordinated pedagogical and administrative approaches in supporting students with diverse learning needs (Mahmood et al., 2025). Collaborative professional learning communities have also been shown to strengthen inclusive practice and may offer a valuable direction for future implementation (Ainscow, 2020).

### Classroom-level recommendations

Differences between elementary and middle school students suggest that inclusion efforts should be sustained and adapted across educational transitions. Middle school students, in particular, may benefit from programs that strengthen social connectedness, reduce stigma, and support increasing academic demands (Eccles et al., 1993). These efforts may also be supported by implementing Multi-Tiered Systems of Support (MTSS), which provide differentiated instruction and targeted interventions to promote equitable participation and success for all learners in general education settings (Howley et al., 2023). While the present findings provide insight into patterns of association, they do not establish causal relationships. Therefore, these practical implications should be interpreted as being consistent with existing literature rather than directly demonstrated by the current study.

### Limitations

This study has several limitations. First, both SLD and perceptions of inclusion were measured using self-report instruments, which may be influenced by social desirability, cultural expectations, or differences in self-awareness (Paulhus and Vazire, 2007). While this introduces potential bias, self-reporting is also a strength of the study given its aim to foreground the perspectives of students themselves, whose voices remain underrepresented in research (Connor and Cavendish, 2020). However, as SLD status was not verified through school records or formal diagnostic documentation, findings should be interpreted as associations between inclusion climate and self-reported indicators of SLD (and reported resource room access), rather than confirmed SLD diagnoses.

Second, the cross-sectional design limits causal interpretation. Longitudinal studies would allow researchers to understand how perceptions of inclusion evolve over time and whether particular educational experiences influence these trajectories (Cohen et al., 2003).

Third, while the translated scales showed good reliability, linguistic, and cultural nuances may influence how students interpret individual items (Hambleton et al., 2005). Future research should explore additional validity evidence and examine whether these measures function similarly across different cultural groups. Including complementary qualitative methods, such as interviews or focus groups, could deepen understanding of students' lived experiences (Creswell and Poth, 2016). Additionally, although confirmatory factor analyses provided initial support for the factor structure of the translated instruments, further research is needed to examine measurement invariance and cross-cultural validity across different populations and contexts.

Fourth, the analytic approach was exploratory and did not account for the nested structure of the data (students within schools). Future research should consider multilevel modeling to account for potential school-level effects and examine how individual and contextual factors jointly relate to perceptions of inclusion.

Fifth, although standardized procedures were used, the availability of assistance during survey completion may have influenced how some students interpreted and responded to items. While this approach supported accessibility, it may have introduced variability in response processes across participants.

Finally, although the inclusion of students from Grades 4 to 9 allowed examination of perceptions across elementary and intermediate school contexts, students within this range differ in developmental stage and educational demands. Future research may examine grade-specific patterns to better understand how perceptions of inclusion change across schooling transitions.

### Conclusion

This study contributes to inclusive education theory by highlighting the importance of student perceptions in understanding inclusion climate, emphasizing that inclusion extends beyond access to encompass belonging and participation. While overall perceptions were positive, lower inclusion among students with higher self-reported indicators of learning disability and among middle school students suggests the need for more responsive and developmentally appropriate inclusive practices. Findings should be interpreted as reflecting associations based on self-reported indicators of learning disability rather than formally diagnosed Specific Learning Disabilities (SLD). The observed relationships were modest in magnitude and should therefore be interpreted with appropriate caution. Overall, findings from the current study emphasize the importance of fostering participation, reducing stigma, and strengthening inclusive classroom practices. These implications are consistent with existing literature and should be considered suggestive rather than directly demonstrated by the present data. Future research should continue to examine how inclusive policies and practices can be strengthened to ensure that all students, regardless of their learning needs, experience supportive, equitable, and inclusive school environments.

## Data Availability

The datasets presented in this article are not readily available because the datasets generated and analyzed during the current study are not publicly available due to the sensitive nature of the participants' information and the ethical requirements to protect student privacy and anonymity. However, the data are available from the corresponding author upon reasonable request and subject to approval by Princess Nourah bin Abdulrhman University institutional ethics committee. Requests to access the datasets should be directed to nfalshalhoub@pnu.edu.sa.
